# Operando analysis of electronic band structure in an all-solid-state thin-film battery

**DOI:** 10.1038/s42004-022-00664-w

**Published:** 2022-04-12

**Authors:** Kazuhiro Hikima, Keisuke Shimizu, Hisao Kiuchi, Yoyo Hinuma, Kota Suzuki, Masaaki Hirayama, Eiichiro Matsubara, Ryoji Kanno

**Affiliations:** 1 Department of Chemical Science and Engineering, School of Materials and Chemical Technology, Tokyo Institute of Technology, 4259 Nagatsuta, Midori-ku, Yokohama, 226-8502 Japan; 2 Department of Electrical and Electronic Information Engineering, Toyohashi University of Technology, 1-1 Hibarigaoka, Tempaku, Toyohashi, Aichi 441-8580 Japan; 3 Research Center for All-Solid-State Battery, Institute of Innovative Research, Tokyo Institute of Technology, 4259 Nagatsuta, Midori-ku, Yokohama, 226-8503 Japan; 4 Office of Society–Academia Collaboration for Innovation, Kyoto University, Kyoto, 611-0011 Japan; 5 Research Institute of Electrochemical Energy, Department of Energy and Environment, National Institute of Advanced Industrial Science and Technology (AIST), 1-8-31, Midorigaoka, Ikeda, Osaka, 563-8577 Japan; 6 Global Base for Nano & Life Innovation Research, Waseda University, Wasedatsurumaki, Shinjuku-ku, 162-0041 Japan

**Keywords:** Batteries, Electronic devices, Energy, Batteries

## Abstract

Material characterization that informs research and development of batteries is generally based on well-established ex situ and in situ experimental methods that do not consider the band structure. This is because experimental extraction of structural information for liquid-electrolyte batteries is extremely challenging. However, this hole in the available experimental data negatively affects the development of new battery systems. Herein, we determined the entire band structure of a model thin-film solid-state battery with respect to an absolute potential using operando hard X-ray photoelectron spectroscopy by treating the battery as a semiconductor device. We confirmed drastic changes in the band structure during charging, such as interfacial band bending, and determined the electrolyte potential window and overpotential location at high voltage. This enabled us to identify possible interfacial side reactions, for example, the formation of the decomposition layer and the space charge layer. Notably, this information can only be obtained by evaluating the battery band structure during operation. The obtained insights deepen our understanding of battery reactions and provide a novel protocol for battery design.

## Abstract (web summary)

The electronic structure evolution within a battery during cycling can provide crucial cues for its optimization, but insights on operando band structures are extremely challenging to obtain. Here, the authors determine the overall band structure of a model thin-film solid-state lithium battery via operando hard X-ray photoelectron spectroscopy, considering the cathode and anode sides.

## Introduction

The voltage of a battery corresponds to the difference in the Fermi levels—or the chemical potentials of the electrons—of the cathode and anode^1^. Scrosati et al. determined the voltage of an intercalation electrode by calculating the changes in thermodynamic energy during the reaction^2^. The thermodynamic behavior of an electrode material depends on its crystal structure, constituent elements, chemical composition, and electronic structure^3^. Based on these properties and characteristics, there are various well-established techniques to prepare and evaluate materials in battery research^4^. These include charge–discharge curve analysis^5^, cyclic voltammetry^6,7^, impedance measurements^8^, X-ray^9^ and neutron^10^ scattering, X-ray absorption near edge structure analysis^11^, and X-ray photoelectron spectroscopy^12–14^. Meanwhile, owing to the importance of the electronic band structure of batteries, several general investigations of battery reactions have been conducted by considering the band structure^15^. However, band structure data are not included in battery analysis or design protocols. Observing the band structures of conventional batteries with liquid electrolytes during operation is usually impossible because conventional batteries with liquid electrolytes are extremely complicated systems. Decomposition of the liquid electrolytes can occur at high voltage, leading to repetitive formation and removal of a solid electrolyte interphase layer with cycling.

In the near future, batteries are expected to undergo a paradigm shift from a liquid-electrolyte configuration, e.g., Li-ion and Pb batteries, to an all-solid-state one, in which all the components, including the electrolyte, are solids^16–19^. Solid-state technology is expected to dramatically improve the capacity, power characteristics, and charging speed of batteries, while avoiding the risks of fire and explosion. The all-solid-state configuration renders the battery a semiconductor device where interfaces of components are junctions of solids stacked in one direction without solid electrolyte interphase layer formation in case of the liquid electrolytes. Thus, hard X-ray photoelectron spectroscopy (HAXPES), which can provide essential data on heterojunction energy level alignment within semiconductors such as diodes and transistors, can be applied to batteries^20^.

**Fig. 1:**
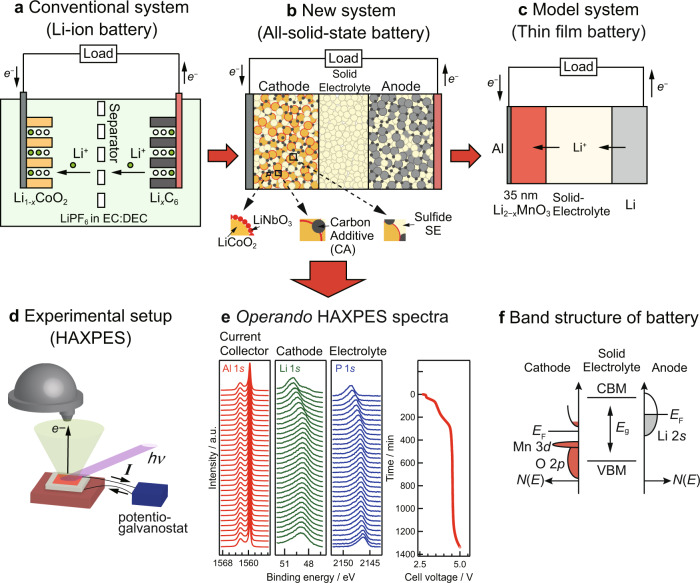
Operando analysis of electronic band structure in all-solid-state batteries. Schematics of **a** prototypical Li-ion battery consisting of intercalation electrodes in a liquid-electrolyte, **b** all-solid-state battery, and **c** the model all-solid-state battery investigated in this study. **d** Experimental setup of operando hard X-ray photoelectron spectroscopy (HAXPES) technique employed to investigate band structure. **e** HAXPE spectra as a function of cell voltage and **f** operando battery band structure analysis. EC ethylene carbonate, DEC diethyl carbonate, CBM conduction band minimum, *E*_F_ Fermi level, VBM valence band maximum.

In this regard, we determined the electronic structure of an all-solid-state battery during charge–discharge cycling using HAXPES. A model all-solid-state thin-film battery was studied to detect electronic structure changes and avoid the various complexities of a practical battery (consisting of powdered materials with coatings and additives; see Fig. 1)^21^. An unrivaled layered oxide, Li_2_MnO_3_, with a theoretical capacity of 459 mAh g^–1^ and maximum practical capacity of 300 mAh g^–1^ ^22–28^, was selected as the cathode. Our previous study suggests that Li_2_MnO_3_ transitions to an activated phase with O1 stacking, with 3D low migration barrier Li diffusion paths, from the original O3 stacking based on in situ XRD measurements and first-principles formation energy calculations^29^. On the other hand, the maximum theoretical capacity of Li_2_MnO_3,_ which is desirable for next-generation battery technologies, has never been realized in practical battery systems.

Our novel approach for the analysis of a model thin-film battery via HAXPES facilitated the elucidation of its band structure; it also revealed drastic band structure changes in the cathode at low voltages along with charge accumulation at the interface at high voltages, thereby preventing the battery from achieving its extremely high theoretical capacity. We also demonstrate how this new method can be used in battery design and for the development of battery protocols.

## Results and discussion

### Energy levels of each component

**Fig. 2:**
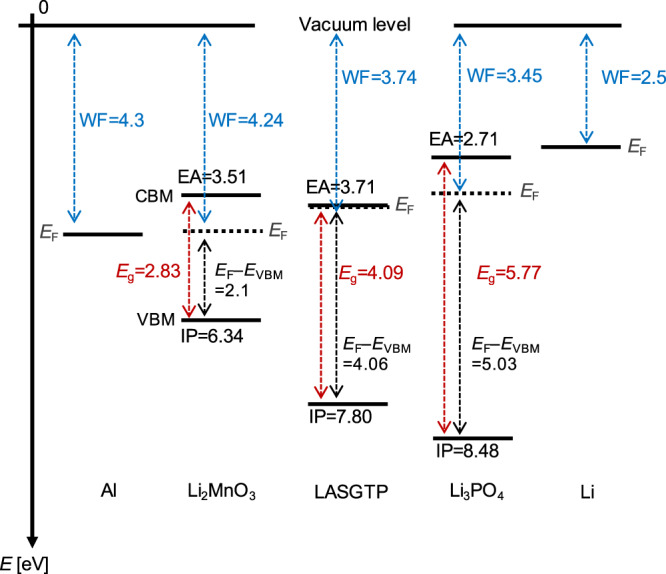
Band edges and Fermi levels of Al, Li_2_MnO_3_, Li_1+*x*+*y*_Al_*x*_(Ti,Ge)_2−*x*_Si_*y*_P_3−*y*_O_12_ (LASGTP), Li_3_PO_4_, and Li aligned to the vacuum level. The work functions (WFs) of Al, Li_2_MnO_3_, LASGTP, Li_3_PO_4_, and Li are shown in blue. The band gaps (*E*_g_) of Li_2_MnO_3_, LASGTP, and Li_3_PO_4_ are shown in red. Electron affinities (EAs) and ionization potentials (IPs) are displayed in black. CBM conduction band minimum, VBM valence band maximum, *E*_F_ Fermi level.

The model all-solid-state battery designed in this study comprised a 10-nm Al current collector, 35.2-nm Li_2_MnO_3_ cathode, 180-μm Li_1+*x*+*y*_Al_*x*_(Ti,Ge)_2−*x*_Si_*y*_P_3−*y*_O_12_ (LASGTP) solid electrolyte, 500-nm Li_3_PO_4_ buffer layer, and 1-μm Li anode^21^. The positions of the bands of the five components with respect to the vacuum level are shown in Fig. 2. The energy levels (vacuum level, conduction band minimum (CBM), Fermi level, and valence band maximum (VBM)) were determined using ultraviolet photoelectron spectroscopy (UPS), low-energy inverse photoemission spectroscopy (LEIPS), and HAXPES, as outlined in the “Methods” section. This information was used as the foundation for subsequent electronic structure analyses. Experimentally obtained work functions were plotted^30,31^ as the Fermi levels for Al^32^ and Li^30^. Using the relative positions of the bands of the bulk components of the battery—the cathode, anode, and electrolyte—the relative locations of the bands at the interface, including data regarding band bending within the depletion layer, were obtained. This information has never been considered in battery development and serves as the starting point of this study. HAXPES and other methods were employed to elucidate the electrode reactions and properties, e.g., the crystal structure, formation and conversion of various redox species, and changes in the valences of the constituent elements during the charge–discharge reaction; these results, obtained via conventional investigations, have been described elsewhere^21,29^.

### Band structure changes of battery

**Fig. 3:**
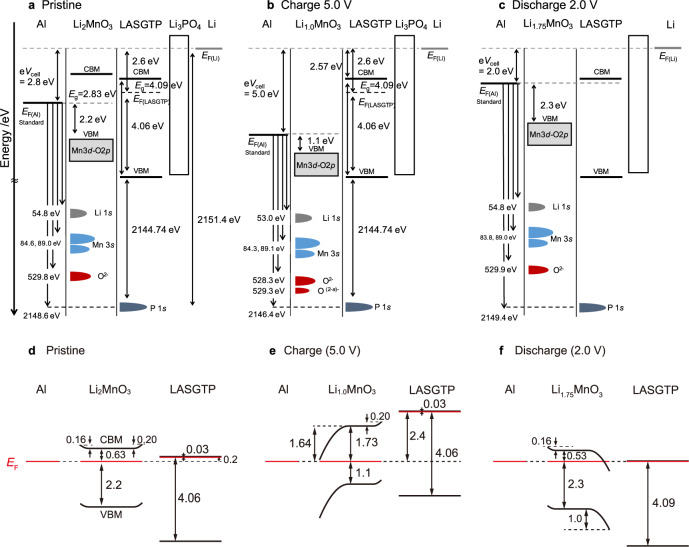
Band structure changes of all-solid-state batteries during cycling. **a**–**c** band diagrams of the battery with minimum assumptions and **d**–**f** band bending of the cathode side with additional assumptions represented by the relative energy level positions, during **a**, **d** pristine, **b**, **e** first charge at 5.0 V, **c**, **f** first discharge at 2.0 V. LASGTP Li_1+*x*+*y*_Al_*x*_(Ti,Ge)_2−*x*_Si_*y*_P_3−*y*_O_12_, CBM conduction band minimum, VBM valence band maximum, *E*_F_ Fermi level, *E*_g_ band gap. Assumptions are detailed in the “Methods” section.

The electronic structure of the model battery during cycling, represented by the relative energy level positions, is shown in Fig. 3 (derivations and assumptions are provided in the “Methods” section). This information is fundamental for analyzing changes in the electronic structure at interfaces owing to the accumulation, depletion, inversion, and formation of electric double layers. The energy level positions of the battery are shown following the first charge and discharge to 5.0 and 2.0 V, respectively. The capacity is 460 mAh g^−1^ when 2Li are extracted from Li_2_MnO_3_, thus capacities of 270 mAh g^−1^ and 200 mAh g^−1^ correspond to cycling of ~1Li and 0.75Li, respectively^21^. We therefore assumed compositions of Li_1.0_MnO_3_ and Li_1.75_MnO_3_ at the first charge and discharge, respectively, according to the charge and discharge capacities. The extent of band bending depends on the relative energies of the vacuum and Fermi levels. The band structure was analyzed by separately considering the cathode (Al/Li_2_MnO_3_/LASGTP) and anode (LASGTP/Li_3_PO_4_/Li) sides.

The Li anode was grounded for the anode (LASGTP/Li_3_PO_4_/Li) analysis; details are explained in the “Methods” section. The Al current collector was grounded for the cathode site analysis. The differences in the Fermi energies between LASGTP and Li was 2.6 eV (i.e., 2151.4 eV [Fermi level (*E*_F_)(Li)–P 1*s*)]–2144.74 eV [VBM(LASGTP)–P 1*s*]–4.06 eV [*E*_F_(LASGTP)–VBM(LASGTP)]) in the cell shown in Fig. 3a. Thus, the energy positions of the LASGTP layer were determined. The Li_2_MnO_3_/LASGTP heterojunction was a staggered gap junction (Type II), with the CBM of Li_2_MnO_3_ being higher than that of LASGTP by 0.2 eV (Fig. 3a, d), as determined from the differences in EA (3.71–3.51 eV), and the VBM of Li_2_MnO_3_ being higher than that of LASGTP by 1.46 eV based on the differences in IP (7.80–6.34 eV) (Fig. 2)^33^. In contrast, Li_2−*x*_MnO_3_ was determined to be inside the potential window (straddling gap heterojunction, Type I) during charging to 5.0 V when band bending was considered (Fig. 3b, e). In addition, the Li_2_MnO_3_/LASGTP heterojunction was a staggered gap junction (Type II) and LASGTP appeared to be outside the potential window by 0.53 eV at 2.0 V when band bending was not considered, indicating that the LASGTP at the interface of the Li_2−*x*_MnO_3_ cathode might undergo reduction during discharge to 2.0 V (Fig. 3c, f).

### Electronic structure changes during battery reaction

**Fig. 4:**
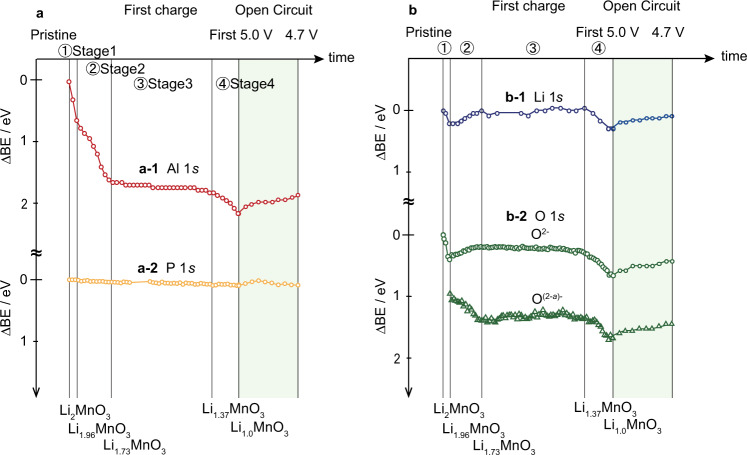
Binding energy shifts (ΔBE) during the first charge to 5.0 V and open-circuit relaxation. ΔBE of **a**, the current collector and electrolyte, **a-1** Al 1s, **a-2** P 1s, and **b** the cathode, **b-1** Li 1*s*, and **b-2** O 1*s* peaks, relative to the Fermi energy of Li during Al cathode-grounded operando hard X-ray photoelectron spectroscopy (HAXPES). Considering the behaviors of the peak shifts in Li 1*s* and O 1*s*, the first charge process can be divided into four stages: stage 1, 2.8 V to 3.25 V (0 ≤ *x* < 0.04 in Li_2−*x*_MnO_3_); stage 2, 3.25 V to 4.38 V (0.04 ≤ *x* < 0.27); stage 3, 4.38 V to 4.65 V (0.27 ≤ *x* < 0.63); and stage 4, 4.65 V to 5.0 V (0.63 ≤ *x* < 1.0). The time scale differs between the charging and open-circuit modes.

Figure 4 shows the shifts in the binding energies of Al, P, Li, and O 1*s* core levels relative to the Fermi energy of Li during the first charge. The Li and O peak positions reflect information on the cathode, whereas the Al and P peak positions reflect the behavior of the current collector and electrolyte, respectively. The P 1*s* core level of the electrolyte does not change significantly with cycling, suggesting that the relative positions of the VBM, Fermi level, and CBM are constant during the first charge, despite Li diffusion through LASGTP.

The binding energy shifts of Li 1*s* and O 1*s* within Li_2_MnO_3_ toward that of Al 1*s* in the current collector and P 1*s* in LASGTP provide information on the electronic structure changes of Li_2_MnO_3_ and its relationship with the LASGTP electrolyte and Al current collector. Shifts in the binding energies of Li and O are caused by the following factors: (1) Differences in the position of the Fermi level of Li_2−*x*_MnO_3_ with respect to that of Al or Li (the metal (Al current collector) and semiconductor (Li_2−*x*_MnO_3_ cathode), which have different work functions as shown in Fig. 2. Electrons move between the metal and the semiconductor to align the *E*_F_ of the semiconductor to the same value as the metal. This means that, ideally, the electrostatic potential of Li_2−*x*_MnO_3_ is similar to that of the Al current collector. (2) Changes in the Fermi level with respect to the VBM, CBM, and core energy levels (e.g., triggered by changes in the carrier density). (3) Changes in Li_2−*x*_MnO_3_ chemistry owing to reactions during cycling, including changes in crystal structure and composition.

The reactions in a battery often cause dramatic changes in structure and composition, and changes in the electronic structure of the cathode material (Li_2−*x*_MnO_3_) are discussed forthwith.

Four stages of binding energy shifts are shown in Fig. 4. We assume compositions of Li_1.96_MnO_3_, Li_1.73_MnO_3_, Li_1.37_MnO_3_ and Li_1.0_MnO_3_ as thresholds of stages 1, 2, 3, and 4 on the basis of charge–discharge capacities 10, 70, 170 and 270 mAh g^−1^, respectively^21^. The binding energies of the Li 1*s* and original O 1*s* peaks (O^2−^) of Li_2−*x*_MnO_3_ shift slightly downwards (increase) with increasing bias voltage at stage 1; the change in bias voltage correlates very well with the shift in the Al 1*s* binding energy. For the cathode side, the Al current collector was grounded to the analyzer and the electronic structure of the Al current collector did not change during charge–discharge reactions, so the shifts of the Al 1*s* binding energy are same as the shifts of *E*_F_ of the analyzer and the Al due to the bias voltage changes. However, at stage 2, the binding energies of the Li 1*s* and original O 1*s* peaks shift slightly upwards (decrease) with increasing bias voltage; additionally, a second O 1*s* peak that shifts downwards is observed. These changes are also observed in a previous study on LiCoO_2_ cathode, showing a peak shift toward lower binding energy at the initial Li deintercalation^34^.

Plateaus are observed at stage 3; during this period charge is extracted while the bias voltage remains almost constant at ~4.6 V. The binding energies of the Li 1*s* and two O 1*s* peaks do not change during stage 3, which means that there are no electronic structure changes in Li_2−__*x*_MnO_3_ during this stage. However, the relative intensities of the O 1*s* peaks do change, with the new peak becoming stronger and the original peak becoming weaker with increasing charging time (Supplementary Fig. 4). This observation is consistent with progression oxygen redox^21^. Finally, as the bias voltage increases to 5.0 V at stage 4, the Li 1*s* and two O 1*s* peaks shift downwards, parallel to the Fermi energy of Al. The parallel shift of the Li 1*s* and two O 1*s* peaks against the Fermi energy of Al means that the electronic structure of the Li_2−__*x*_MnO_3_ do not change during stage 4.

### Discussion

Changes that occur during the four stages are initiated in either the electrode, electrolyte, or at the electrode–electrolyte interface. The changes during stages 1 and 2 indicate that minor Li extraction causes drastic changes in the electronic structure of the cathode, as observed for the first time in this study using operando HAXPES.

During stage 1, the Fermi level of Li_2−*x*_MnO_3_ shifted downwards drastically, whereas the other levels remained almost constant. We proposed structural changes with cycling, which is irreversible transition from the original O3 to the O1 stacking, with better Li-ion diffusion characteristics, that can proceed only at a high state-of-charge at a low *x* (*x* < 1.25)^32^. This happens during stage 4 in this paper. Similar relative differences were assumed to exist between the CBM, VBM, and core energy levels of Li_2_MnO_3_ and Li_1.96_MnO_3_, because their crystal structures are almost the same^32^. In addition, Supplementary Fig. 3 shows a comparison of the energy levels of the battery in the pristine state (2.8 V) and after the first charge to 3.25 V (composition: Li_1.96_MnO_3_). The Li 1*s* and O 1*s* levels shifted downwards by 0.14 and 0.11 eV, respectively, with the alignment of the Li Fermi level, as shown in Supplementary Fig. 3. Therefore, the VBM and CBM can be understood to have shifted downwards by ~0.1 eV during charging to 3.25 V (shown in blue). The Fermi energy of Li_2−*x*_MnO_3_ appeared to be identical to that of Al, and therefore the Fermi level of Li_1.96_MnO_3_ was considered to have shifted downwards by 0.45 eV (corresponding to the shift in the Fermi level of Al (3.25–2.8 eV)), which is ~0.32 eV (0.45–0.14 eV (Li 1*s* shift), shown in blue) larger than the almost constant shifts of the other levels (Li 1*s*, O 1*s*, VBM, and CBM shown in Supplementary Fig. 3).

During stage 2, a transition from an n-type to a p-type semiconductor was observed. The Fermi level of Al gradually shifted downwards by ~0.8 eV as the composition shifted from Li_1.96_MnO_3_ to Li_1.73_MnO_3_ (Fig. 4a). Assuming a constant band gap of 2.8 eV, a total Fermi level downward shift of 1.1 eV (2.2–1.1 eV in Fig. 3d, e, determined using Supplementary Fig. 11) with respect to the VBM caused a transition from an n-type to a p-type semiconductor. The fact that the Fermi level drops to the level of the VBM during the first charge (Supplementary Fig. 3) was impossible to deduce from the analyses of the band structures and binding energies of the original crystal and electronic structures, which is observed at the first time.

A transition from an n-type to a p-type semiconductor means that the Ohmic contact at the initial state becomes a Schottky contact at the Al/Li_2−*x*_MnO_3_ interface in the middle of the charge–discharge reaction. This change interferes with the charge–discharge reaction due to the rectifying property at the Al/Li_2−*x*_MnO_3_ interface. Consequently, an inversion layer appeared at the Al/Li_2−*x*_MnO_3_ interface at high voltage, because the Al/Li_2−*x*_MnO_3_ interface is n-type and the Li_2−*x*_MnO_3_/LASGTP interface is p-type, as shown in Fig. 3e, which means that there is the rectifying property of electrons in the Li_2−*x*_MnO_3_ cathode. This electronic structure could show that the current flows only from the LASGTP solid electrolyte to the Al current collector side (p to n direction), which indicated that only discharging is possible. In other words, charging cannot proceed at high voltage. This is consistent with the experimental results, which slight charge capacity can be obtained at high voltage.

Stage 3 involved a change in the binding energy mainly owing to compositional changes associated with (de)intercalation within the cathode because of the slight changes of their crystal structures at stage 3^32^. This has been explained in another study^21^.

Stage 4 featured an overvoltage, the location of which was confirmed via HAXPES. With increasing bias voltage after the plateau region, the Al Fermi level, Li 1*s* peak, two O 1*s* peaks shifted downwards in parallel. Although the crystal structure transitions from O3 to O1 stacking at this stage, the interpretation of this phenomenon is difficult^32^. Charging was stopped at 5.0 V and the battery was relaxed under open-circuit conditions to understand this behavior. The results during relaxation clearly indicate that the potential of Li_2−*x*_MnO_3_ was not immediately relaxed and the changes during charging from 4.6 to 5.0 V are mostly reversible, indicating that Li_2−*x*_MnO_3_ at 5.0 V may be far from thermodynamic equilibrium. Bulk and interface contributions could have been responsible for the deviation from thermal equilibrium. A higher overpotential and/or electronic conductivity might have been necessary to overcome the activation barrier in bulk Li_2−*x*_MnO_3_. Ion displacement and charge accumulation resulting in a potential gradient would occur at bulk Li_2−*x*_MnO_3_ if the Li_2−*x*_MnO_3_ electrode is an insulator with a certain dielectric constant. In this case, the ion displacement and charge accumulation would be reversed when the potential gradient disappears. There was no peak broadening of O 1*s* at Li_2−*x*_MnO_3_ based on the experimental depth profiling results (Supplementary Fig. 12), indicating there is no potential gradient in bulk Li_2−*x*_MnO_3_. The activation barrier in bulk Li_2−*x*_MnO_3_ could play the minor role at the stage 4.

Another possibility involves the reversible formation of a capacitor region at the Li_2−*x*_MnO_3_/LASGTP interface, and not across the entire volume of Li_2−*x*_MnO_3_ or LASGTP, above 4.6 V. Intrinsic changes in the electronic structure or the stoichiometry of Li_2−*x*_MnO_3_ or LASGTP do not occur if the battery acts as a capacitor; therefore, each component experiences the same internal potential except at the interface. The voltage at the capacitor region at the interface increased with charge accumulation, and the relationship was linear at constant capacitance. Electrochemical reactions involving Li deintercalation did not occur during this stage, despite the large potential gap at the electrode/electrolyte interface. Therefore, the reversible formation of a capacitor region at the Li_2−*x*_MnO_3_/LASGTP interface could play the main role at stage 4.

The reaction mechanism for the extremely high-capacity cathode material Li_2_MnO_3_ was hereby clarified. Reversible redox reaction with oxygen proceeded at 2–4.5 V, whereas no Li (de)intercalation occurred in the high-voltage region with capacitor-like behavior at the Li_2_MnO_3_/LASGTP interface. To achieve cathode capacities closer to the theoretical limit of 459 mAh g^–1^, we propose further modification of the system by (1) increasing ion diffusion in the cathode material or interface layer, (2) increasing electrical conductivity by electron or hole doping of the electrode, or (3) supplying electrons directly to the charge transfer reaction sites at the interface.

## Conclusions

An experimental method for observing battery band structures, similar to the approach used for the analyses of semiconductor heterostructures, was developed. The electronic structure within a battery during cycling, which is critical for analyzing the internal reactions, was established for the first time using a fabricated model thin-film all-solid-state Al/Li_2−*x*_MnO_3_/LASGTP/Li_3_PO_4_/Li battery and operando HAXPES. The obtained band schemes provided insight into the Li_2−*x*_MnO_3_ reaction mechanisms. Details such as the potential window of the electrolyte, overvoltage, and extent of band bending at the Al/Li_2−*x*_MnO_3_ and Li_2−*x*_MnO_3_/LASGTP interfaces were obtained at the pristine state (Figs. 2 and 3a, d). In addition, a transition of Li_2−*x*_MnO_3_ from an n-type to a p-type semiconductor and features of overvoltage were observed at the initial and end charge state, respectively, for the first time based on operando HAXPES measurements results during battery reaction (Figs. 3b, c, e, f and 4). This type of data should be incorporated into general protocols for battery design and battery material development, and we have presented an example of how the data we obtained can be used for battery design. This will allow the quantitative design of electrode–electrolyte interfaces (which previously were designed by trial and error) such as interface modifications, combinations of the electrodes/electrolytes and/or current correctors/electrodes to be realised using novel methods similar to those used for analyzing semiconductors. However, additional considerations would be necessary when discussing all-solid-state batteries with thick cathode and anode, because this study is limited to exploration of an all-solid-state thin-film battery.

## Methods

### Battery fabrication

The all-solid-state thin-film batteries used in this study consisted of five components: an Al current collector, Li_2_MnO_3_ cathode, LASGTP solid electrolyte substrate, Li_3_PO_4_ buffer layer, and Li anode (Fig. 1c). The Li_2_MnO_3_ film was synthesized via pulsed laser deposition using a 248-nm KrF excimer laser (COMPex 201, Coherent, Santa Clara, CA, USA). The synthesis conditions were as follows: substrate temperature = 773 K, target-to-substrate distance = 60 mm, laser frequency = 5 Hz, deposition time = 10 min, laser energy = 1.1 J cm^–2^, and oxygen pressure = 75 Pa^21^. An LASGTP glass ceramic sheet (9.8 mm × 9.8 mm × 180 μm, Ohara, Sagamihara, Japan) was employed as the substrate^35^. The composition of the Li_2_MnO_3_ film was controlled by the target composition and oxygen pressure, with a sintered pellet (20 mm diameter × 3 mm height, Toshima Manufacturing, Saitama, Japan) of Li_2.4_MnO_3_ (20 mol% Li excess) used as the target. Al film was deposited on the Li_2_MnO_3_ film via electron beam deposition. A ~500-nm-thick Li_3_PO_4_ buffer layer was synthesized via radio-frequency sputtering (PSAD-3000, AOV, Tokyo, Japan) on the LASGTP substrate, on the side opposite to that of the Li_2_MnO_3_ film. Stoichiometric Li_3_PO_4_ targets (Toshima Manufacturing, Saitama, Japan) were held 30 mm away from the substrate. Li_1+*x*_Al_*x*_Ti_2−*x*_(PO_4_)_3_, which is a compound related to LASGTP, undergoes a redox reaction where Ti^3+^ transforms into Ti^4+^ when the cell voltage is reduced below 2.5 V (vs. Li/Li^+^)^36^. Li_3_PO_4_ has a wide band gap and enables achievement of stable charge–discharge reactions at the all-solid-state Li metal batteries, thus a Li_3_PO_4_ buffer layer was introduced to prevent any reaction between LASGTP solid electrolyte and Li metal^21,37^.

Li film was deposited on the Li_3_PO_4_ film as the counter electrode via vacuum deposition (E-80Li, ALS Technology Co., Ltd., Kanagawa, Japan). All the steps involved in the synthesis of the all-solid-state battery assembly were conducted at 298 K in an Ar-filled glove box with O_2_ < 1.0 ppm and H_2_O < 0.1 ppm to prevent reactions of the Li metal and Li_3_PO_4_ buffer layer with moisture and CO_2_ present in the air.

### Thin-film characterization

The thin films were characterized via grazing incidence X-ray diffraction (XRD) using a thin-film X-ray diffractometer with Mo Kα radiation (SmartLab, Rigaku, Tokyo, Japan). The thicknesses, densities, and roughness of the films were determined via X-ray reflectivity (XRR) measurements using an X-ray diffractometer with Cu Kα_1_ radiation (ATX-G, Rigaku). Cross-sectional scanning transmission electron microscopy (STEM) and energy dispersive X-ray (EDS) analysis were conducted using a JEM-2010 microscope (JEOL, Tokyo, Japan). The structural characteristics of the Li_2_MnO_3_ thin-film on the LASGTP substrate have been reported previously^21^. The XRD patterns verified the growth of Li_2_MnO_3_ film on the LASGTP substrate. The thickness and surface roughness of the Li_2_MnO_3_ film on the LASGTP substrate were 35.2 and 1.1 nm, respectively, according to the XRR analysis. Cross-sectional STEM images and EDS maps of the Li_2_MnO_3_ film on the LASGTP substrate have been published in another of our studies^21^.

### Ultraviolet photoelectron spectroscopy (UPS)

UPS was performed with an incident light energy of 21.22 eV (He I source), path energy of 1.3 eV, and step size of 0.01 eV, using a PHI 5000 VersaProbe III (ULVAC-PHI, Chigasaki, Japan). A bias of −5 V was applied to the sample to measure the secondary electron cut-off energy. Spectra were also obtained with zero bias to measure the VBM edge.

### Low-energy inverse photoemission spectroscopy (LEIPS)

LEIPS was conducted using an electron gun (1 μA and 20 V) mounted on the PHI 5000 VersaProbe III. The band-pass filters were 4.77 eV (260 nm/16 nm full width at half maximum (FWHM)) or 3.70 eV (335 nm/7 nm FWHM), and the step size was 0.04 eV. Low-energy electron transmission (LEET) spectra were simultaneously obtained using a microcurrent meter.

### Operando hard X-ray photoelectron spectroscopy (HAXPES)

HAXPES was conducted on both sides of the LASGTP substrate at the SPring-8 BL28XU beamline using a hemispherical electron energy analyzer (R4000, Scienta Omicron, Uppsala, Sweden) with an incident photon energy level of ~7940 eV and a photoelectron take-off angle of 89°^38^. The escape depth of photoelectrons with an incident photon energy of ~6900 eV is ~47 nm while the probing depth of standard Al Kα X-ray sources is limited to several nanometers^14^. Therefore, photoelectrons can penetrate the Al current collector adsorbed on the Li_2_MnO_3_ cathode film, enabling collection of data on the surface, bulk Li_2_MnO_3_, and Li_2_MnO_3_/LASGTP interface by an incident photon energy level of using ~7940 eV. Details of the peak fitting have been provided elsewhere^21^. Operando HAXPES was performed at a current density of 0.467 μA cm^–2^ (0.1 C), using constant current charge and discharge mode. Battery cells were placed in a sample holder within an Ar-filled glove box and carefully transported to a vacuum chamber without exposure to air/moisture. The spectrum corresponding to Al was subtracted from the raw data because the valence band spectrum included a contribution from the Al current collector. Examples of raw (uncalibrated) data for the first discharge are shown in Supplementary Fig. 4.

Two grounding options, namely, grounding of the working electrode (Al cathode) and the counter electrode (Li anode), were considered. As shown in Supplementary Fig. 5, the as-observed Al 1*s* peaks appeared at an almost constant binding energy, and the binding energy of the P 1*s* peak, which mostly corresponds to the LASGTP substrate, exhibited a linear correlation with the applied voltage (slope: 1 eV/V) when the Al current collector was grounded (left panel). In contrast, the binding energy of the P 1*s* peak was almost constant, and that of the Al 1*s* peak exhibited a linear correlation with the applied voltage (slope: 1 eV/V) when the Li anode was grounded (right panel). In both grounding scenarios, the *E*_F_ positions of the Al current collector shifted with the applied cell voltage, leading to the shifts in the binding energies, as observed in previous studies^39^. The relative peak positions were identical, and hence the more convenient grounding was adopted in subsequent analyses; the Al current collector was grounded for the cathode (Al/Li_2_MnO_3_/LASGTP) analysis and the Li anode was grounded for the anode (LASGTP/Li_3_PO_4_/Li) analysis.

### Band edge determination

The determination of the band edges is explained using Li_2_MnO_3_ as an example. Supplementary Fig. 6 shows UP spectra obtained at a bias voltage of −5 V. The edges of the VBM and secondary electrons are two critical parameters; these edges were obtained through linear extrapolation to the horizontal axis (zero intensity). The VBM edge was at −2.9 eV and the Fermi energy was −5 eV (Supplementary Fig. 6), which corresponds to the bias voltage; therefore, the VBM was 2.1 eV below the Fermi energy. Conversely, the secondary electron edge, which corresponds to the maximum secondary electron energy, was 11.98 eV (Supplementary Fig. 6), which is 14.88 eV below the VBM. The VBM was therefore 21.22 − 14.88 = 6.34 eV below the vacuum level. The IP is defined as follows:

$${{{{{\rm{IP}}}}}}=h\nu -({E}_{{{{{{\rm{SE}}}}}}\_{{{{{\rm{edge}}}}}}}-{E}_{{{{{{\rm{VBM}}}}}}}),$$

where $h\nu$ is the incident light energy of UPS measurement (21.22 eV in this study), and *E*_VBM_ and *E*_SE_edge_ are the binding energies of the VBM and secondary electron edge, respectively.

The LEIP and LEET spectra are shown in Supplementary Fig. 6. The EA was calculated as follows:

$${{{{{\rm{EA}}}}}}={E}_{{{{{{\rm{BP}}}}}}}-({E}_{{{{{{\rm{onset}}}}}}}-{E}_{0}),$$

where *E*_BP_ is the band-pass filter energy (4.77 eV in this study), *E*_onset_ is the onset energy in the LEIP spectrum, and *E*_0_ is the inflection point in the LEET spectrum. *E*_onset_ was obtained through linear extrapolation, as for the UP spectrum, while *E*_0_ represents the local maximum of the derivative of the LEET spectrum. The energies of *E*_0_ and *E*_onset_ were found to be −7.6 and −6.34 eV, respectively; therefore, the EA of Li_2_MnO_3_ was determined to be 4.77 − (−6.34 − (−7.6)) = 3.51 eV. The UP and LEIP spectra of Li_2_MnO_3_ are plotted together in Supplementary Fig. 6.

The band edges for LASGTP (Supplementary Fig. 7) and Li_3_PO_4_ (Supplementary Fig. 8) were obtained in a similar manner.

### Band bending rules

Band bending between two components, A and B, was subsequently considered, as shown in Supplementary Fig. 1. The extent of band bending depends on the relative energies of the vacuum and Fermi levels (*E*_vac_ and *E*_F_ in Supplementary Fig. 1, respectively) and the difference in the Fermi levels when the vacuum level is aligned. The relationships *a* + *c* = *b* and *a* + *f* + *g* + *h* = *b* are evident in Supplementary Fig. 1. When the energy difference between Fermi levels changed from *c* to *f*, the total extent of band bending (*g* + *h*) was obtained as *c* − *f*. However, it was not possible to obtain the relative amounts of *g* and *h* and the width of the depletion layer without further information, such as the carrier density and dielectric constant. The energies differences between the vacuum level and other bands remained identical when a junction was formed (i.e., *d* and *e* did not change with junction formation). Consequently, the extent of the band bending of the vacuum and other energy levels in a component, including the VBM and CBM, were similar.

### Determination of electronic structures shown in Fig. 3a–c

#### Anode (LASGTP/Li_3_PO_4_/Li)

The energy differences between the P 1*s* peak and the Fermi level of Li, and the P 1*s* peak and the VBM of LASGTP, were 2151.4 and 2144.74 eV, respectively (Supplementary Figs. 7 and 9). Therefore, the energy difference between the Fermi level of the Li and VBM of the LASGTP was 6.66 eV. In addition, the energy difference between the Fermi level of the LASGTP and the VBM of LASGTP was 4.06 eV, as shown in Fig. 2; therefore, the Fermi energy difference between LASGTP and Li was 2.6 eV. Thus, the LASGTP band positions in the cell were obtained by evaluating the binding energy of the P 1*s* peak during charge and discharge when Li or Al was grounded (Supplementary Fig. 10), in combination with analysis of the UP and LEIP spectra (Supplementary Fig. 7). The Fermi energy difference between LASGTP and Li was 2.6 eV in the cell and 1.24 eV in the respective materials aligned to the vacuum level, resulting in a net band bending of 1.36 eV (see band bending rules in Supplementary Fig. 1). However, if the Fermi levels of Li_3_PO_4_ and Li were set to be equal, net band bending values of 2.60 and 0.95 eV were exhibited by the LASGTP/Li_3_PO_4_ and Li_3_PO_4_/Li interfaces in the opposite directions, respectively (Supplementary Fig. 2). If this extent of band bending did not occur, the Fermi level shifted with respect to the band edge positions of Li_3_PO_4_, for example, from very slight changes in the Li^+^ concentration (an example featuring a Fermi level shift to the CBM is shown in Supplementary Fig. 2) and/or formation of an interface layer at the Li_3_PO_4_/Li interface. This value did not change during the first charge, based on the assumption that the relative positions of the VBM, Fermi level, and CBM were constant, despite Li diffusion through LASGTP.

#### Cathode (Al/Li_2_MnO_3_/LASGTP)

The electronic structure of the cathode (Al/Li_2_MnO_3_/LASGTP) changed with the voltage, and the state at battery assembly was initially considered. The open-circuit voltage in the assembled state was 2.8 V, according to electrochemical measurements, indicating that the Fermi level of the Li anode was 2.8 eV higher than that of the Al current collector^15,40^. The core energy levels in Li_2_MnO_3_, such as Li 1*s*, Mn 3*s*, and O 1*s*, were obtained with respect to the Al Fermi level via HAXPES. The energy levels of the VBM and core energy levels were similarly obtained when the battery was cycled, using the HAXPE spectra shown in Supplementary Fig. 1. However, the relative positions of the VBM, Fermi level, and CBM obtained using the UP and LEIP spectra in Fig. 2 could not be employed because the crystal structure of Li_2−*x*_MnO_3_ during cycling was not similar to that of pristine Li_2_MnO_3_. Therefore, information on the Fermi level and CBM of Li_2−*x*_MnO_3_ is absent from Fig. 3b, c. In addition, the Fermi level of LASGTP is not shown in the first discharge to 2.0 V. If the Fermi level of LASGTP is set to 2.6 eV below the Fermi level of Li, it is no longer between those of the Li anode and Al cathode. This suggests that the bias voltages at the interfaces would no longer be unidirectional. The Fermi level of LASGTP was therefore expected to be somewhere between those of the Li anode and Al cathode, i.e., outside the band gap, assuming no shift of the VBM and CBM with respect to the vacuum level. Li_1+*x*_Al_*x*_Ti_2−*x*_(PO_4_)_3_, which is a compound related to LASGTP, undergoes a redox reaction, with Ti^3+^ being transformed into Ti^4+^ when the cell voltage is reduced below 2.5 V (vs. Li/Li^+^)^36^. This value is extremely close to the Fermi level of LASGTP and approaches the Fermi level of Li in the pristine state, which is 2.6 V. Therefore, the Fermi level of LASGTP was deduced to change position when the voltage was below ~2.6 V, possibly due to the Ti redox reaction.

### Evaluation of band bending as shown in Fig. 3d–f with additional assumptions

Figure 3d–f shows the estimated electronic band diagrams of Al/Li_2−*x*_MnO_3_/LASGTP featuring the VBM, Fermi level, and CBM. The experimental results obtained herein facilitated the construction of these diagrams, although several additional assumptions were required for the purpose comprehensiveness.

(1) The Fermi level of Li_2−*x*_MnO_3_ during cycling was identical to that of Al, i.e., the bias voltage at the Al/Li_2−*x*_MnO_3_ interface can be neglected. This is a relatively safe assumption; however, it is inappropriate if a substantial bias voltage exists at the Al/Li_2−*x*_MnO_3_ interface, for example, due to the formation of an insulating layer. Similarly, the Fermi level of Li_2−*x*_MnO_3_ at the first discharge (2.0 V) was identical to that of Al (Fig. 3d–f).

(2) The energy difference between the vacuum level and Li 1*s* core level in Li_2−*x*_MnO_3_ during cycling was similar to that of Li_2_MnO_3_ in the pristine state. This assumption involves similar crystal structures and local environments around Li and no changes in the valency of Li during cycling of Li_2−*x*_MnO_3_. The core levels of Mn could not be used because of changes in the valency of Mn during cycling and a possible intrinsic shift due to these changes. Likewise, the energy difference between the vacuum level and P 1*s* core level was assumed to be similar to that of LASGTP during cycling.

(3) The band gap of Li_2−*x*_MnO_3_ did not change during cycling. This is a considerably broad assumption. The VBM is at the top of the valence band. A qualitative approach suggests that the VBM of Li_2−*x*_MnO_3_ becomes deeper with cycling because electrons at the top of the valence band are removed upon cycling, resulting in transformation into empty states. The base of the empty states becomes the CBM. However, the CBM is not at the bottom of the vacated states in the original valence band. This was clear from the difference of 1.1 eV between the Fermi level and VBM in Li_2−*x*_MnO_3_ after charging to 5.0 V (Fig. 3b). Therefore, we assumed that the vacated states shifted to the bottom of the conduction band and the band gap was constant. The prioritization of an increase or decrease in the band gap during cycling was challenging.

(4) The VBM and CBM positions of LASGTP with respect to the P 1*s* core level changed to accommodate the Fermi level within the band gap at 2.0 V. A major change in stoichiometry or crystal structure was not anticipated within LASGTP during cycling; therefore, the band gap remained constant. The Fermi level was set at the CBM to minimize shifts in the VBM and CBM; however, the total extent of band bending did not depend on the positions of the VBM and CBM.

(5) Band bending at the Li_2−*x*_MnO_3_/LASGTP interface was assumed to occur entirely on the Li_2−*x*_MnO_3_ side. This is because no band bending was observed within LASGTP in preliminary HAXPES of the P 1*s* core level with different take-off angles. However, only the total amount of band bending on both sides of the interface could be determined based on the analysis of energy differences between the vacuum level, CBM, Fermi level, and VBM (see Derivation of band bending in Methods). The limit of a flat band practically corresponds to an infinitely large carrier density on one side, such as that at a metal/semiconductor interface. Although LASGTP is not a metal, Li can diffuse within LASGTP and thereby possesses the ability to alter the carrier density.

The extents of the band bending shown in Fig. 3d–f are derived below.

#### A. Pristine Al/Li_2_MnO_3_ (Fig. 3d)

The WF (= *E*_vac_ − *E*_F_) of Al was 4.3 eV (Fig. 2) and the VBM of Li_2_MnO_3_ was 2.2 eV below the Fermi level of Al (Fig. 3a). Using the IP of Li_2_MnO_3_ obtained via LEIPS measurements only (6.34 eV, Fig. 2), and assuming that the Fermi levels of Al and Li_2_MnO_3_ were similar, the WF of Li_2_MnO_3_ was calculated as 6.34 − 2.2 = 4.14 eV. Using the relationship obtained by considering the band bending, the band bending was calculated to be 4.3 − 4.14 = 0.16 eV.

#### B. Pristine Li_2_MnO_3_/LASGTP (Fig. 3d)

The WF of LASGTP was 3.74 eV (Fig. 2) and the VBM of LASGTP was 4.06 eV below the Fermi level of LASGTP (Fig. 3a). Using the IP of LASGTP obtained using LEIPS measurements only (7.8 eV, Fig. 2), the WF of LASGTP was calculated to be 7.8 – 4.06 = 3.74 eV. The difference in the WFs of Li_2_MnO_3_ (section A) and LASGTP, which represents the difference between the Fermi levels aligned at the vacuum level, was 4.14 – 3.74 = 0.4 eV. However, the difference in Fermi levels at the interface of Li_2_MnO_3_ and LASGTP within the battery was 2.8 − 2.6 = 0.2 eV. The band bending was calculated as 0.40 − 0.20 = 0.20 eV using the band bending rules.

#### C. First charge (5.0 V) of Al/Li_1.0_MnO_3_ (Fig. 3e)

The WF of Al was 4.3 eV (Fig. 2), and the energy difference between the vacuum level of Li and the Li 1*s* core energy level was assumed to be constant during cycling. In the pristine state, the energy difference corresponded to the sum of the WF of Li_2_MnO_3_ and the binding energy of the Li 1*s* core energy level with respect to the Fermi level of Al (the Fermi level of Li_2_MnO_3_), i.e., 4.14 + 54.8 = 58.94 eV (Fig. 3a). In the first charge state, the energy difference between the vacuum level of Li and the Fermi level of Li_1.0_MnO_3_ was 58.94 − 53.0 = 5.94 eV (Fig. 3b), which is also considered to be the WF of Li_1.0_MnO_3_. The difference between the WFs of Li_1.0_MnO_3_ and Al was 5.94 − 4.3 = 1.64 eV, which was the extent of the band bending.

#### D. First charge (5.0 V) of Li_1.0_MnO_3_/LASGTP (Fig. 3e)

The relative positions of the vacuum level, Fermi level, and P 1*s* core levels were assumed to be constant during cycling; consequently, the WF of LASGTP remained constant at 3.74 eV (Fig. 3b). Therefore, the difference in the WFs at the interface was 5.94 – 3.74 = 2.20 eV. Moreover, under the same assumption, the difference in Fermi levels at the interface within the battery was calculated to be 5.0 − 2.6 = 2.4 eV (Fig. 3b), indicating that the band bending was 2.4 – 2.2 = 0.20 eV, as per the band bending rules.

#### E. First discharge (2.0 V) of Al/Li_1.75_MnO_3_ (Fig. 3f)

Similar to the observations noted in section C, the Li vacuum level was 58.94 − 54.8 = 4.14 eV higher than the Fermi level of Al, which is also considered to be the Fermi level of Li_1.75_MnO_3_ (Fig. 3c). The band bending was 4.3 − 4.14 = 0.16 eV.

#### F. First discharge (2.0 V) of Li_1.75_MnO_3_/LASGTP (Fig. 3f)

A reverse bias would be expected to appear if the relative positions of the energy levels (vacuum level, CBM, Fermi level, VBM, and P 1*s* core level) were constant during cycling, i.e., the Fermi level of LASGTP would be lower than that of Al in such a case. To avoid this issue, the Fermi level of LASGTP was allowed to shift so as to be similar to those of Al and Li_1.75_MnO_3_ and the CBM of LASGTP. The difference in Fermi levels at the interface was therefore zero. The difference between the P 1*s* core level and vacuum level of LASGTP was 2144.74 + 7.80 = 2152.54 eV (Fig. 3a); therefore, the WF of LASGTP from Fig. 3c was 2152.54 – 2149.4 = 3.14 eV. Finally, the difference between the WFs of Li_1.75_MnO_3_ and LASGTP was 4.14 – 3.14 = 1.0 eV, which is the band bending at Li_1.75_MnO_3_/LASGTP interface.

## Supplementary information

Supplementary Information

## Data Availability

The datasets generated during the current study are available from the corresponding author upon reasonable request.
