# Supplementary material for: Operando analysis of electronic band structure in an all-solid-state thin-film battery
Source: Commun Chem. 2022 Apr 12;5:52. doi: 10.1038/s42004-022-00664-w (PMC9814885; doi:10.1038/s42004-022-00664-w)
Supplement: Supplementary file 1 — Supplementary Information [file 42004_2022_664_MOESM1_ESM.docx]

**Supplementary Information**

**Operando analysis of electronic band structure in an all-solid-state thin-film battery**

*Kazuhiro Hikima^1,2^, Keisuke Shimizu^3^, Hisao Kiuchi^4^, Yoyo Hinuma^3,5^, Kota Suzuki^3^, Masaaki Hirayama^1,3^, Eiichiro Matsubara^6^, and Ryoji Kanno^3*^*

^1^Department of Chemical Science and Engineering, School of Materials and Chemical Technology, Tokyo Institute of Technology, 4259 Nagatsuta, Midori-ku, Yokohama 226-8502, Japan

^2^Department of Electrical and Electronic Information Engineering, Toyohashi University of Technology, 1-1 Hibarigaoka, Tempaku, Toyohashi, Aichi 441-8580, Japan

^3^Research Center for All-Solid-State Battery, Institute of Innovative Research, Tokyo Institute of Technology, 4259 Nagatsuta, Midori-ku, Yokohama 226-8503, Japan

^4^Office of Society–Academia Collaboration for Innovation, Kyoto University, Kyoto 611-0011, Japan

^5^Research Institute of Electrochemical Energy, Department of Energy and Environment, National Institute of Advanced Industrial Science and Technology (AIST), 1-8-31, Midorigaoka, Ikeda, Osaka 563-8577, Japan

^6^Global Base for Nano & Life Innovation Research, Waseda University, 513, Wasedatsurumaki, Shinjuku-ku, Tokyo 162-0041, Japan.

***Corresponding author:** Ryoji Kanno (E-mail: kanno@echem.titech.ac.jp)

**Supplementary Fig. 1** | Differences between energy levels used to determine the extent of band bending. *E*_F_, Fermi level; *E*_VAC_, relative energy of the vacuum level; VBM, valence band maximum.

**Supplementary Fig. 2** **|** Band bending within the anode assuming that **a**, relative energy differences between the valence band maximum (VBM), conduction band minimum (CBM), and Fermi level (*E*_F_) do not change with battery assembly and **b**, *E*_F_ of Li_3_PO_4_ shifts to the CBM to minimise band bending. The extent of band bending is split equally between the two sides at the Li_1+_*_x_*_+_*_y_*Al*_x_*(Ti,Ge)_2−_*_x_*Si*_y_*P_3−_*_y_*O_12_ (LASGTP)/Li_3_PO_4_ interface and is entirely contained within Li_3_PO_4_ at the Li_3_PO_4_/Li interface.

**Supplementary Fig. 3** | Key features of hard X-ray photoelectron spectra near the P 1*s* peak of the Al/Li_1+_*_x_*_+_*_y_*Al*_x_*(Ti,Ge)_2−_*_x_*Si*_y_*P_3−_*_y_*O_12_ (LASGTP) interface obtained when **a**, Li and **b**, Al are grounded. The energy difference between the Fermi level (*E*_F_) and valence band maximum (VBM) within LASGTP is obtained via ultraviolet photoelectron spectroscopy. CBM, conduction band minimum; *E*_g_, band gap.

**Supplementary Fig. 4** | Uncalibrated **a**, **e**, Al 1*s*, **b**, **f**, O 1*s*, and **c**, **g**, Mn 3*s* operando hard X-ray photoelectron spectra of the Li_2_MnO_3_ cathode on a Li_1+_*_x_*_+_*_y_*Al*_x_*(Ti,Ge)_2−_*_x_*Si*_y_*P_3−_*_y_*O_12_ substrate during **a**–**c**, first charge and **e**–**g**, first discharge. The **d**, first charge and **h**, first discharge capacity–voltage curves are also shown.

**Supplementary Fig. 5** | **Two types of hard X-ray photoelectron spectroscopy.** Comparison of the Al 1*s* and P 1*s* spectra, voltage curves, and differences in binding energies (BE) as a function of voltage when **a**, Al and **b**, Li are grounded. Measurements are conducted during the first discharge, and BEs correspond to the pristine state (battery assembly, 2.8 V). CE, counter electrode; WE, working electrode; LASGTP, Li_1+_*_x_*_+_*_y_*Al*_x_*(Ti,Ge)_2−_*_x_*Si*_y_*P_3−_*_y_*O_12_.

**Supplementary Fig. 6** | **Ultraviolet photoelectron (UP) spectra of Li_2_MnO_3_ with a bias voltage of −5 V.** **a**, Entire spectrum, **b**, near the secondary electron edge, and **c**, near the valence band maximum (VBM). **d**, Low-energy inverse photoemission (LEIP) spectrum, low-energy electron transmission (LEET) spectrum, and the derivative of the LEET spectrum of Li_2_MnO_3._ **e**, Enlarged view of the LEIP spectrum near the onset energy. **f**, UP and LEIP spectra shown with the vacuum level (V.L.), conduction band minimum (CBM), Fermi level (*E*_F_), and VBM. IP, ionisation potential; *E*_BP_, band-pass filter energy; EA, electron affinity; *E*_g_, band gap; *E*_0_, LEET spectrum inflection point.

**Supplementary Fig. 7** | **Ultraviolet photoelectron (UP) spectra of Li_1+_*_x_*_+_*_y_*Al*_x_*(Ti,Ge)_2−_*_x_*Si*_y_*P_3−_*_y_*O_12_ (LASGTP) with a bias voltage of** **−5 V. a**, Entire spectrum, **b**, near the secondary electron edge, and **c**, near the valence band maximum (VBM). **d**, UP spectra of LASGTP near the VBM with a zero-bias. **e**, Low-energy inverse photoemission (LEIP) spectrum, low-energy electron transmission (LEET) spectrum, and the derivative of the LEET spectrum of LASGTP_._ **f**, Enlarged view of the LEIP spectrum near the onset energy. **g**, UP and LEIP spectra shown with the vacuum level (V.L.), conduction band minimum (CBM), Fermi level (*E*_F_), and VBM. IP, ionisation potential; *E*_BP_, band-pass filter energy; EA, electron affinity; *E*_g_, band gap; *E*_0_, LEET spectrum inflection point.

**Supplementary Fig. 8** | **Ultraviolet photoelectron (UP) spectra of Li_3_PO_4_ with a bias voltage of −5 V:** **a**, the entire spectrum, **b**, near the secondary electron edge, and **c**, near the valence band maximum (VBM). **d**, Low-energy inverse photoemission (LEIP) spectrum, and low-energy electron transmission (LEET) spectrum, and the derivative of the LEET spectrum of Li_3_PO_4_. **e**, Enlarged view of the LEIP spectrum near the onset energy. **f**, UP and LEIP spectra shown with the vacuum level (V.L.), conduction band minimum (CBM), Fermi level (*E*_F_), and VBM. IP, ionisation potential; *E*_BP_, band-pass filter energy; EA, electron affinity; *E*_g_, band gap; *E*_0_, LEET spectrum inflection point.

**Supplementary Fig. 9** | **Derivation of the Li_1+_*_x_*_+_*_y_*Al*_x_*(Ti,Ge)_2−_*_x_*Si*_y_*P_3−_*_y_*O_12_ (LASGTP) P 1*s* core energy level in the Al/LASGTP**. **a**, Hard X-ray photoelectron spectrum with Al grounded. **b**, Relationship between the P 1*s* level of LASGTP and *E*_F_ of Al.

**Supplementary Fig. 10** | **Derivation of the Li_1+_*_x_*_+_*_y_*Al*_x_*(Ti,Ge)_2−_*_x_*Si*_y_*P_3−_*_y_*O_12_ (LASGTP) P 1*s* core energy level in the Al/Li_2_MnO_3_/LASGTP/Li_3_PO_4_/Li cell**. Hard X-ray photoelectron spectrum after the first charge and discharge when **a**, Li and **b**, Al are grounded, respectively. **c**, Relationship between the P 1*s* level of LASGTP and Fermi level (*E*_F_) of Li after the first charge and discharge. Relationship between the P 1*s* level of LASGTP and *E*_F_ of Al after the **d**, first charge and **e**, first discharge.

**Supplementary Fig. 11 |** Al-grounded hard X-ray photoelectron spectra of Li_2−_*_x_*MnO_3_ near the Al Fermi level in an Al/Li_2_MnO_3_/Li_1+_*_x_*_+_*_y_*Al*_x_*(Ti,Ge)_2−_*_x_*Si*_y_*P_3−_*_y_*O_12_/Li_3_PO_4_/Li battery during cycling **a**, of the battery assembly, **b**, at first charge to 5.0 V, and **c**, at first discharge to 2.0 V. The valence band maximum of Li_2−_*_x_*MnO_3_ is obtained via linear extrapolation of the edge to zero intensity.

**Supplementary Fig. 12** | **Depth profiling results of hard X-ray photoelectron spectroscopy**.

Uncalibrated O 1*s* hard X-ray photoelectron spectra of the Li_2_MnO_3_ cathode on a Li_1+_*_x_*_+_*_y_*Al*_x_*(Ti,Ge)_2−_*_x_*Si*_y_*P_3−_*_y_*O_12_ substrate when Li is grounded with TOA of 88.8°, 60°, and 30°. Measurements are conducted during the first charge. TOA, Take Off Angle.
